# The role of altered decision dynamics and dorsolateral prefrontal cortex to amygdala causal circuitry in the aberrant efficacy of emotion suppression in subthreshold depression

**DOI:** 10.1017/S0033291725103097

**Published:** 2026-01-30

**Authors:** Lijing Niu, Timothea Toulopoulou, Xiaoqi Song, Qian Li, Haowei Dai, Keyin Chen, Jiayuan Zhang, Xiayan Chen, Zini Chen, Xingqin Wang, Delong Zhang, Ruibin Zhang

**Affiliations:** 1 Cognitive Control and Brain Healthy Laboratory, Department of Psychology, School of Public Health, Southern Medical University, Guangzhou, China; 2 Department of Psychology, Department of Neuroscience, National Magnetic Resonance Research Center (UMRAM) & Aysel Sabuncu Brain Research Center, Bilkent University, 06800 Ankara, Turkey; 3 1st Department of Psychiatry, National and Kapodistrian University of Athens, Athens, Greece; 4 Department of Psychiatry, Icahn School of Medicine at Mount Sinai, New York, USA; 5 Department of Neurosurgery, Institute of Brain Diseases, Nanfang Hospital of Southern Medical University, Guangzhou, China; 6 Key Laboratory of Brain, Cognition and Education Sciences (South China Normal University), Ministry of Education, Guangzhou 510631, China; School of Psychology, Center for Studies of Psychological Application, and Guangdong Key Laboratory of Mental Health and Cognitive Science, South China Normal University, Guangzhou 510631, China; 7 Department of Psychiatry, Zhujiang Hospital, Southern Medical University, Guangzhou, PRC China; 8 Guangdong-Hong Kong-Macao Greater Bay Area Center for Brain Science and Brain-Inspired Intelligence, Guangdong-Hong Kong Joint Laboratory for Psychiatric Disorders, Guangdong Basic Research Center of Excellence for Integrated Traditional and Western Medicine for Qingzhi Diseases, Southern Medical University, China

**Keywords:** drift diffusion model, dynamic causal modeling, emotion suppression, subthreshold depression

## Abstract

**Background:**

Individuals with subthreshold depression (StD), a potentially preclinical stage of major depression, may habitually employ maladaptive expression suppression strategies in emotion regulation. However, the effect of emotional suppression (EES) and underlying neural mechanisms remain unclear.

**Methods:**

Data came from two samples (Sample 1: 55 StD, 60 healthy controls (HC); Sample 2: 23 StD, 20 HC). Both samples completed expression suppression tasks. Using drift diffusion modeling, we decomposed performance on the emotional assessment process into separate processing components, particularly the speed of information update (drift rate), to examine how depression and emotional suppression affect decision-making. To further reveal the potential mechanism, we conducted fMRI scanning in Sample 2 and characterized latent neurocircuit driving emotion suppression and drift rate using dynamic causal modeling (DCM).

**Results:**

The EES negatively correlated with drift rate. StD showed reduced efficacy of EES and faster drift rates of negative preference. Greater activation was observed in the dorsolateral prefrontal cortex (dlPFC) and amygdala in StD during suppression. DCM analysis revealed that inefficient EES might be explained by the stronger connection from the right dlPFC to the right amygdala, while the faster drift rate might be attributed to a stronger connection from the left amygdala to the right dlPFC.

**Conclusions:**

Our study uncovered novel latent behavioral and neurocircuit mechanisms of early risk for depression. Ineffective emotional suppression in StD is associated with faster accumulation of negative evidence. The underlying neural mechanism may involve aberrant regulation between the dlPFC and amygdala in negative contexts.

## Introduction

Emotion suppression, a response-focused strategy prevalent in various mental disorders, is characterized by a decrease in emotional expression behaviors without alterations to the underlying negative emotional experience (Gross, 2002). Studies have indicated that emotional preferences can skew the decision-making process toward preferred options (Lerner, Li, Valdesolo, & Kassam, 2015). When confronted with emotional events, depressed patients often resort to maladaptive suppression strategies due to a greater inclination to exhibit negative emotions in situation selection and attention allocation (Vanderlind, Millgram, Baskin-Sommers, Clark, & Joormann, 2020). However, this suppression strategy does not reduce negative emotions. Moreover, according to prior research, habitual suppression is linked to higher levels of negative emotional experiences (Koval, Butler, Hollenstein, Lanteigne, & Kuppens, 2015) and anhedonia (Werner-Seidler, Banks, Dunn, & Moulds, 2013), and it is a key factor contributing to the difficulty individuals with depressive disorders have in maintaining a positive mood (Vanderlind et al., 2020).

The drift diffusion model (DDM) is a computational model (Peters & D’Esposito, 2020) used to dissociate observed behavior into latent dynamic processes that represent distinct cognitive affective components, including initial bias (*β*; priori preference), drift rate (*δ*; the rate of evidence accumulation), decision threshold (*α*; the amount of evidence required), and non-decision time (*τ*) (Forstmann, Ratcliff, & Wagenmakers, 2016; Warren et al., 2020). The DDM can better detect differences among subject populations than reaction time or accuracy alone (Forstmann et al., 2016); for instance, DDM application successfully revealed that children with anxiety show impairments in multiple latent behavioral dynamic measures, including initial bias toward positive and higher drift rates under the reappraisal than the aversive condition (Warren et al., 2020). Meanwhile, subliminal affective priming studies have revealed that individuals with greater levels of fear tend to accumulate negative facial information more rapidly, reflected in the existence of a higher drift rate toward negative target words (Kim, Kim, Kim, & Jeong, 2021). This implies that emotional preferences, such as fear, can modulate the speed and direction of evidence accumulation in decision-making tasks, thereby influencing the rate at which participants drift from *X* (e.g. neutral) to *Y* (e.g. negative). In other words, drift rates may represent changes in emotional preferences.

To achieve goals that either increase or decrease emotional responsivity, control related regions modulate subcortical (amygdala, ventral striatum) and cortical (insula) emotion systems by up-regulating or down-regulating their activity (Sikka, Stenberg, Vorobyev, & Gross, 2022). During depressive states, the brain’s processing of negative information often involves an overactive bottom–up system, like an overactivated amygdala. Simultaneously, a compromised inhibitory function of the top–down system, such as the dorsolateral prefrontal cortex (dlPFC), may intensify this process. This could potentially create a maladaptive feedback loop that might lead to a depressive episode (Zhengzhi et al., 2015). Lopez et al. found that decreased dlPFC activity in response to negative stimuli is pivotal in depression (Lopez, Denny, & Fagundes, 2018). A systematic review indicated that, when compared to simply watching and reacting naturally, actively suppressing the outward expression of emotions when exposed to negative stimuli increases activation in areas of the frontoparietal control network, with the right dlPFC particularly involved in expressive suppression (Sikka et al., 2022). Increased amygdala activation is often associated with negative emotions, such as fear and disgust (Sergerie, Chochol, & Armony, 2008). Gao et al. documented stronger dlPFC–amygdala connectivity in individuals who often use suppression strategies (Gao, Biswal, Chen, Wu, & Yuan, 2021), but the directionality was not clear.

Subthreshold depression (StD) refers to the existence of relevant depressive symptoms in individuals, but the number, severity, and duration of symptoms do not meet the diagnostic criteria of a major depressive episode (Rodríguez, Nuevo, Chatterji, & Ayuso-Mateos, 2012), which is the prodromal stage (Cuijpers & Smit, 2004) and an important risk indicator (Eaton, Badawi, & Melton, 1995) of depression. Although the symptoms of StD are milder than those of depression, it can also lead to many serious consequences, such as decreased quality of life (Nierenberg et al., 2010), dysfunction (Karsten, Penninx, Verboom, Nolen, & Hartman, 2013), and an increased risk of suicide (Balázs et al., 2013). The prevalence of StD is high, and its incidence is usually higher than that of depression (Cuijpers et al., 2013). Moreover, individuals with StD have a greater chance of developing depression than healthy controls (HC) (Jinnin et al., 2017). With the worsening of depression, an individual’s increasing lack of hedonic experience becomes more noticeable (McIntosh, Sullivan, & Lewis, 2019). Individuals with StD, even when experiencing the same positive emotions as individuals without depression, were unable to maintain a positive mood after the positive stimuli disappeared. This trend is similar to what occurs in depression (Song et al., 2024). Individuals with depression and those in remission from depressive disorder were also found to be more likely to hold back their emotions and to use more strategies to suppress their expressions when compared to HC. For instance, research has indicated that frequent suppression and fewer positive experiences are linked to anhedonia.

In StD, it remains uncertain how the use of suppression strategies impacts emotional experiences, decision-making, and the underlying neural mechanisms. Herein, the first aim of this study was to extend the existing knowledge on the impact of emotional suppression on emotional experience in StD through laboratory studies and to explore its computational behavioral mechanisms using the DDM. Second, we conducted task-based functional magnetic resonance imaging (fMRI) studies to examine the effects of emotional suppression on the brain mechanisms in StD. For this aim, we used the same emotional suppression paradigm adopted to satisfy the first aim, along with a dynamic causal model (DCM) to study the suppression of neural connection structure changes. Using the DCM enables us to model directional neuronal connectivity and understand how it generates the observed BOLD signal during emotional activity (Friston, Harrison, & Penny, 2003).

## Methods

### Participants

#### Sample 1

Sample 1 participants completed the experiment in a laboratory setting. Students were recruited from universities in Guangzhou, China, and screened using a two-stage procedure (Li et al., 2015; Takagaki et al., 2014). In the first stage, we used both the Beck Depression Inventory-II (BDI-II) and the Center for Epidemiological Studies Depression Scale (CES-D) to identify potential StD candidates based on established cutoffs. All participants completed both the BDI-II and the CES-D. In line with prior research (Gotlib, McLachlan, & Katz, 1988; Li et al., 2015), BDI scores of ≤6 points indicated that an individual was not depressed, while a score of ≥14 points indicated minor depression. On the CES-D, a score of 16 points was the critical threshold point for depression (Radloff, 1977). Participants who scored ≥14 points on the BDI-II and ≥16 points on the CES-D were invited to the second stage of screening.

**Figure 1.**
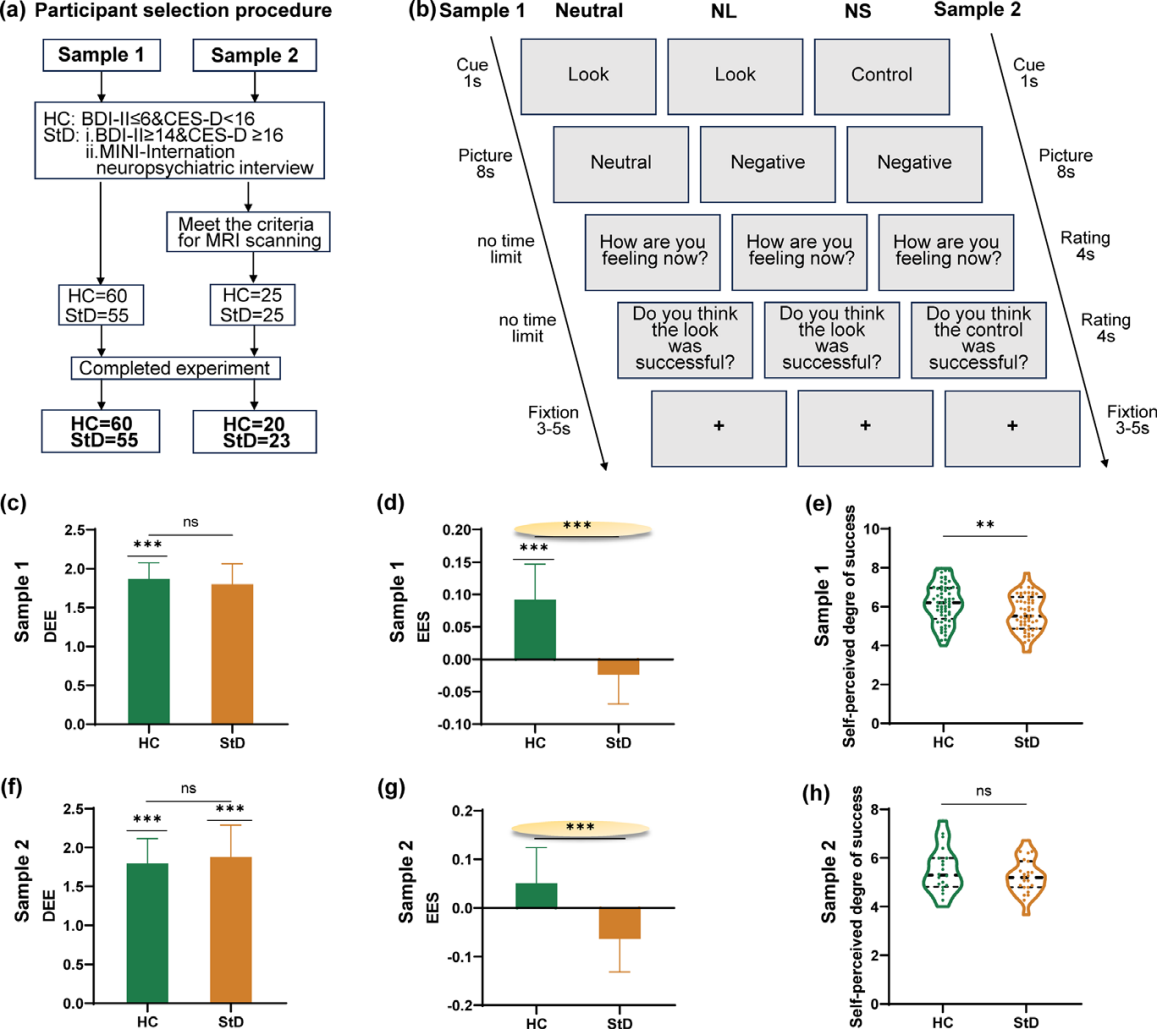
**Schematic view of participant selection, emotion suppression task and participant performance. (a)** The participant selection procedure outlines the criteria and steps for recruiting and screening individuals suitable for the sample 1(HC:60, StD:55) and sample 2 (HC:20, StD:23). **(b)** The emotion suppression task part illustrates the specific tasks performed by participants. Based on cue words (1 second), participants viewed images (neutral/negative) for 8 seconds and engaged in different psychological operations. For the LOOK cues, they experienced the emotion induced by the image. For the Control cues, they suppressed the emotion evoked by the picture. After the picture disappeared, participants monitored their emotional state and self-perceived operational success (study1: no time limit; study2: 4 seconds). The next trial began after a 3-5 second fixation period. Study1 included 90 trials, while study2 included 45 trials. **(c)** Degree of Emotional Elicitation (DEE) in Sample 1. StD experienced similar levels of negative emotions as HC when presented with negative stimuli. **(d)** Effect of Emotional Suppression (EES) in Sample 1. After suppressing negative emotions, the negative emotions of HC significantly decreased, whereas the emotions of StD remained largely unchanged. The EES for StD was significantly worse than for HC. **(e)** Self-perceived degree of success in Sample 1. Both groups had average success scores greater than 5. **(f)** Degree of Emotional Elicitation (DEE) in Sample 2. **(g)** Effect of Emotional Suppression (EES) in Sample 2. **(h)** Self-perceived degree of success in Sample 2. The results of Sample 2 were consistent with those of Sample 1.

In the second stage, we administered the Mini International Neuropsychiatric Interview (MINI) to rule out diagnosable psychiatric disorders, particularly current or past major depressive disorder (MDD), and to verify that participants showed at least one core depressive symptom (depressed mood or anhedonia) without meeting full MDD criteria. Participants underwent a structured clinical evaluation using the M.I.N.I. conducted by two trained postgraduate students in the Department of Psychology. Based on this two-stage procedure, Sample 1 finally included 55 StD participants and 60 HC (Figure 1a) (refer to the Supplementary Material for details for detailed inclusion and exclusion criteria). This study involving human participants was reviewed and approved by the Institutional Review Board of Southern Medical University.

#### Sample 2

Participants completed the experiment while undergoing fMRI scanning in Sample 2. In addition to meeting the inclusion criteria of Sample 1, all participants had to meet the MRI-specific inclusion criteria (Supplementary Material) for inclusion in Sample 2. Sample 2 finally included 23 StD and 20 HC (Figure 1a). The participants in Sample 2 were independent of the participants in Sample 1.

### Self-assessment questionnaire

All self-report measures were administered using validated Chinese versions of the original scales. In addition to the BDI-II (Beck & Brown, 1996) and the CES-D (Radloff, 1977), participants were required to complete the Self-Rating Anxiety Scale (Zung, 1971) and the Trait Anxiety Inventory (T-AI) (Spielberger et al., 1983) to assess their levels of anxiety. Furthermore, the Emotion Regulation Questionnaire (Gross & John, 2003) was administered to measure the use of cognitive reappraisal and expressive suppression strategies. In both samples, significant differences in anxiety and depression levels were observed between the two groups (Table 1).

**Table 1.** Self-assessment questionnaire data

	StD		HC		*t*	*p*
	Mean	Std.	Mean	Std.		
*Sample 1*						
BDI-II	22.41	7.56	2.13	1.81	−20.17	<.001
CES-D	29.89	8.63	5.92	4.59	−18.82	<.001
SAS	38.20	5.96	32.18	5.43	−5.67	<.001
T-AI	49.69	6.15	40.58	7.11	−7.32	<.001
ERQ						
Cognitive reappraisal	29.09	6.09	33.38	5.75	3.89	<.001
Expressive suppression	14.64	5.59	12.65	4.92	−2.03	.045
*Sample2*						
BDI-II	22.87	7.18	1.50	1.32	−13.11	<.001
CES-D	31.04	9.23	4.25	3.17	−12.34	<.001
SAS	38.17	4.84	33.70	3.39	−3.46	<.001
T-AI	47.74	3.69	44.45	4.39	−2.68	.011
ERQ						
Cognitive reappraisal	29.13	5.35	31.70	6.17	1.46	.151
Expressive suppression	15.30	5.63	12.70	4.28	−1.69	.099

*Note:* BDI-II, Beck Depression Inventory-II; CES-D, Center for Epidemiological Studies Depression Scale; ERQ, emotion regulation questionnaire; SAS, self-rating anxiety scale; T-AI, trait anxiety inventory.

### Experimental design and task paradigm

The emotion suppression task used in this study follows a well-established paradigm for assessing suppression as an emotion-regulation strategy (Gross & Levenson, 1993; Ochsner et al., 2004). This suppression paradigm has also been successfully applied in studies of individuals with anxiety and depressive symptoms, confirming its sensitivity to emotion-regulation deficits in both subclinical and clinical populations (Vanderhasselt, Kühn, & De Raedt, 2013; Warren et al., 2020). Pictures were chosen from the International Affective Picture System (Bradley & Lang, 1994), and we re-rated the valence and arousal to finalize 60 negative and 30 neutral images for Sample 1 (see the Supplementary Material and Table S1 for details of the experimental material). For Sample 2, 30 negative and 15 neutral images were randomly chosen from Sample 1’s set (Supplementary Table S1).

During the experimental stage of the study, participants completed consent forms and questionnaires, received instructions, practiced with four trials, and then began the formal experiment. Participants were told that, during each trial, they would first see an instructional cue word (Look or Control), followed by an image and then two self-report rating scales, and the last is looking at fixation (Figure 1b). For ‘Control’ cues, participants were asked to suppress the emotional response elicited by the picture. This is similar to the so-called ‘poker face’, where they try not to give anyone insight into their emotional world. This approach that has been validated in prior work on expressive suppression (Bebko, Franconeri, Ochsner, & Chiao, 2011). For ‘Look’ cues, participants were asked to demonstrate their feelings toward the picture. The first self-report question rated emotional state from 1 to 4 point(s) (very negative to negative) or 5–8 points (positive to very positive), while the second assessed the success of the Look/Control strategy, rating it from 1 to 4 point(s) (very unsuccessful to unsuccessful) or 5–8 points (successful to very successful) (Supplementary Material). Participants scored their emotional state under the following three conditions. Neutral, ‘Look’ followed by a neutral image. NL, ‘Look’ followed by a negative image. NS, ‘Control’ followed by a negative image. There were 30 trials under each of the conditions in Sample 1.

The task paradigm in Sample 2 was consistent with that in Sample 1. With necessary adaptations for the fMRI setting, the presentation time for the two rating scales was standardized to 4 s. There were 45 trials in Sample 2.

### Behavioral index

Participant ratings of the first self-report served as a behavioral index of the degree of emotion elicitation (DEE) and the effect of emotional suppression (EES). The DDE used the following equation:
(1)

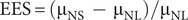

with higher scores indicating greater emotion elicitation.

Separately, EES was computed using the following equation:
(2)

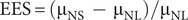

with higher scores indicating more effective emotional suppression.

We first conducted one sample *t* tests to assess the DEE and EES within each group. Subsequently, we performed two-sample *t* tests to examine the differences between the two groups.

The second self-report score under the NS condition was used to measure the extent to which participants believed they were successful in suppressing their emotions (self-perceived emotional-suppression success). If the average score was >5 points, suppression was considered successful.

### Computational modeling of latent behavioral dynamics

**Figure 2.**
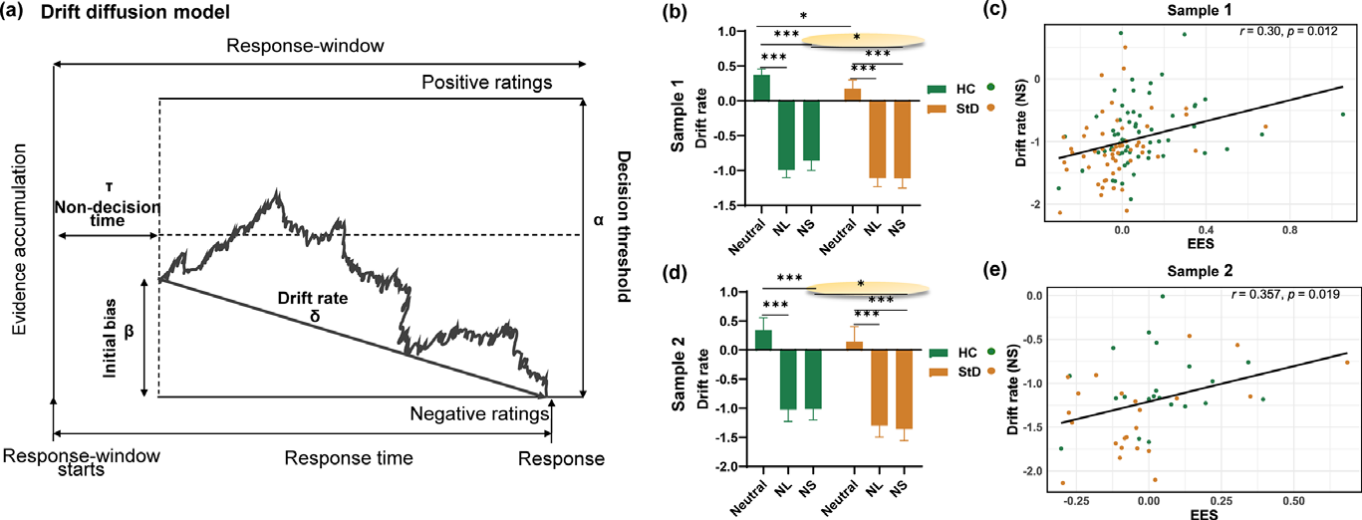
**Drift diffusion model and participant performance. (a)** Illustration of a single trial of the drift diffusion process, demonstrating how noisy evidence of positive and negative stimulus evaluations accumulates over time in a random walk. During each trial, participants experience an initial deviation captured in an eight-second window, reflecting their initial positive or negative response to the stimulus(*β*). This serves as the starting point for the random walk. The drift rate(*δ*) represents the rate at which evidence accumulates during the response window, influencing the direction and speed of the random walk towards decision boundaries(*α*). Decision boundaries, separated by a decision threshold, mark points at which a response is made based on accumulated evidence. **(b)** Participants of both groups in Sample 1 showed significant differences in drift rate (*δ*) in the Neutral and NS condition. StD assessed their emotions as positive more slowly during neutral context and assessed their emotions as negative more quickly after attempting to suppress negative emotions. **(c)** Correlation Between EES and Drift Rate (NS) in Sample 1. There was a positive correlation between the EES and drift rate in the NS condition. Poorer EES was associated with a faster rate of rating emotions as negative after suppression. **(d)** Participants of both groups in Sample 2 showed significant differences in drift rate (*δ*) in the NS condition. **(e)** Correlation Between EES and Drift Rate (NS) in Sample 2. HC: Healthy control; StD: Subthreshold depression; Neutral: Neutral picture with the cue word “Look”; NL: Negative picture with the cue word “Look”; NS: Negative picture with the cue word “Control”.

The emotion evaluation process was modeled as a drift-diffusion process, in which evidence accumulates over time, resulting in a decision when a decision threshold is reached. The evaluations were coded as negative (ratings of 1–4 points) or positive (ratings of 5–8 points) (Figure 1b). The initial bias (*β*) represented the starting point for the drift-diffusion process, and it captured the initial reaction during image viewing, before the decision window. The drift rate parameter (*δ*) characterizes evidence accumulation, with higher values indicating a more rapid determination of emotional experience; the direction of the *δ* indicates the tendency of emotional preference, with a positive *δ* signifying a greater inclination to perceive the emotional experience as positive, while a negative *δ* indicates the opposite. Finally, the decision threshold parameter (*α*) captures response caution, or the degree of confidence required to conclusively evaluate emotion, with higher values characterizing slower and more consistent responses. Meanwhile, the non-decision time (*τ*), reflecting perceptual processes before evidence accumulation, for each individual was fixed across instructions and stimulus types (Figure 2a). Computational modeling was performed in the Docker HDDM framework (https://hub.docker.com/r/hcp4715/hddm/tags) (Hu et al., 2022) (see the Supplementary Material for details of DDM analysis).

To examine the differences between conditions within each group and the differences between groups for each condition, two-way analysis of variance (ANOVA) was conducted for each parameter. Post-hoc analyses were conducted using Tukey’s honest significant difference (HSD) test to identify specific differences between conditions within each group for the significant effects found in the ANOVA. Additionally, correlation analyses were conducted between DDM parameters, EES, and questionnaire scores to further investigate the underlying psychological processes involved in individual decision-making.

### Computational modeling of dynamic causal interactions

All imaging data were collected on a 3-T Trio Tim scanner (Siemens, Munich, Germany) with a 64-channel phased-array head coil in the Brain Imaging Center of South China Normal University. Task-functional fMRI data were preprocessed using Statistical Parametric Mapping software (SPM12; http://www.fil.ion.ucl.ac.uk/spm/software/spm12/). For each participant, we first conducted general linear model (GLM) analysis to identify the significant differences in brain activation between the two groups (see the Supplementary Material for details of imaging data acquisition, preprocessing, and GLM analysis).

To analyze causal interactions between brain regions, we performed a DCM analysis using SPM12. We determined the individual activation peak points for each participant around the peaks of activation in bilateral dlPFC and amygdala regions obtained from group-level analyses, which are frequently reported as key nodes for facilitating emotion regulation in classic theories. Then, using the peak voxel of each participant’s corresponding brain region as the center and a radius of 8 mm, we defined the four regions of interest for each participant (Table 2).

**Table 2.** The MNI coordinates of the four VOIs used as nodes in the DCM analysis

VOI	MNI coordinates		
	x	y	z
dlPFC_L	−9	42	42
dlPFC_R	12	42	45
Amygdala_L	−20	0	−21
Amygdala_R	24	−3	−21

*Note:* dlPFC, dorsolateral prefrontal cortex; _L, left hemisphere; _R, right hemisphere.

The network specification in DCM was set up to model the entire experiment using three matrices(Zeidman et al., 2019), including (i) matrix A, where fixed connections existed between the regions; (ii) matrix B, where modulatory connections existed; and (iii) matrix C, which encompasses the driving input to the system. We established full connectivity in the matrix in which all regions were connected. Matrix B demonstrates how much the strength of the connection changes due to different experimental conditions (Figure 4a).

A parametric empirical Bayes (PEB) framework was used when testing the individual differences in behavioral performance explained by the difference in brain connectivity (Zeidman et al., 2019). There were five regressors in the PEB design matrix, including (1) the group mean of connectivity strength, (2) the EES, (3) the *δ* (NS), (4) the CES-D scores, and (5) the BDI scores, and we were particularly interested in the EES and the *δ* (NS) among them. We next performed a post-hoc analysis known as auto-search Bayesian model reduction (BMR) to ascertain whether any modulatory effect in matrix B did not contribute to the model fitness. To ensure the robustness of the PEB estimation, we set a threshold of model parameters at a posterior probability of >95% (strong evidence of the parameters being present rather than absent) in the auto-search BMR. Notably, in the task-based time-domain DCM, as implemented in SPM12, estimated effective connectivity parameters were expressed in units of Hz. Here, Hz denotes the rate constant of neuronal influence between regions (i.e. coupling strength per unit time), rather than oscillatory frequency. This convention is standard in DCM (Friston et al., 2003).

## Results

### Sample 1

#### Reduced efficacy of emotion suppression effects in StD

In Sample 1, participants rated their emotional reaction to three task conditions. The rating scores were significantly lower in the NL condition than in the Neutral condition (HC: *t*_59_ = 18.07, *p* < .001; StD: *t*_54_ = 13.52, *p* < .001; Figure 1c). In particular, the results showed that the negative stimuli successfully induced negative emotions in participants, without a significant difference between the two groups (*t*_113_ = 0.42, *p* = .678; Figure 1c). The EES in the StD group was significantly worse than that in the HC group (M_HC_ = 0.09, M_StD_ = −0.02, *t*_113_ = 3.32, *p* = .002; Figure 1d). Both groups had average success scores of >5 points, indicating that participants in both groups believed they were successful in inhibiting their emotions (M_HC_ = 6.16, M_StD_ = 5.67, *t*_113_ = 2.71, *p* = .008; Figure 1e).

#### Faster drift rates of negative preference in StD

The two-way ANOVA for *δ* revealed significant main effects of group (*F*_(1,113)_ = 14.463, *p* < .001) and condition (*F*_(2,113)_ = 300.303, *p* < .001). However, the interaction effect between group and condition was not significant (*F*_(2,113)_ = 0.589, *p* = .556). For HC, the post-hoc test revealed significant differences between the Neutral versus NL (mean difference [MD] = 1.358, *p* < .001), and Neutral versus NS (MD = 1.229, *p* < .001) conditions. Similarly, for StD participants, the post-hoc test showed significant differences between the Neutral versus NL (MD = 1.283, *p* < .001) and Neutral versus NS (MD = 1.287, *p* < .001) conditions. More importantly, given the significant main effects found in relation to group, the independent-samples *t* test found significant differences between the two groups under the Neutral (Neutral: *M*_HC_ = 0.369, *M*_StD_ = 0.176, *t*_113_ = 2.588, *p* = .011; Figure 2b) and NS (NS: *M*_HC_ = −0.859, *M*_StD_ = −1.111, *t*_113_ = 2.577, *p* = .011; Figure 2b) conditions. See the Supplementary Material for details of the ANOVA results for *α*, *β*, and *τ.*

Additionally, the correlation analysis results showed that EES was positively associated with *δ* (*r* = 0.30, *p* = .012; Figure 2c) and negatively correlated with *α* in NS (*r* = −0.246, *p* = .008; Supplementary Figure S1b).

### Sample 2

The next goal was to explore the neural mechanisms underlying the poorer EES and faster *δ* in StD participants. To this end, we recruited new participants again and completed the experiment while conducting MRI scanning. Considering task performance, the poorer EES (*M*_HC_ = 0.51, *M*_StD_ = −0.06, *t*_41_ = 2.36, *p* = .023; Figure 1g) and faster *δ* for the NS condition (*M*_HC_ = −1.01, *M*_StD_ = −1.36, *t*_41_ = 2.61, *p* = .013; Figure 2d) seen among Sample 1 StD participants were replicated in Sample 2 (Supplementary Material).

#### Differences in brain activation of emotion suppression between groups

**Figure 3.**
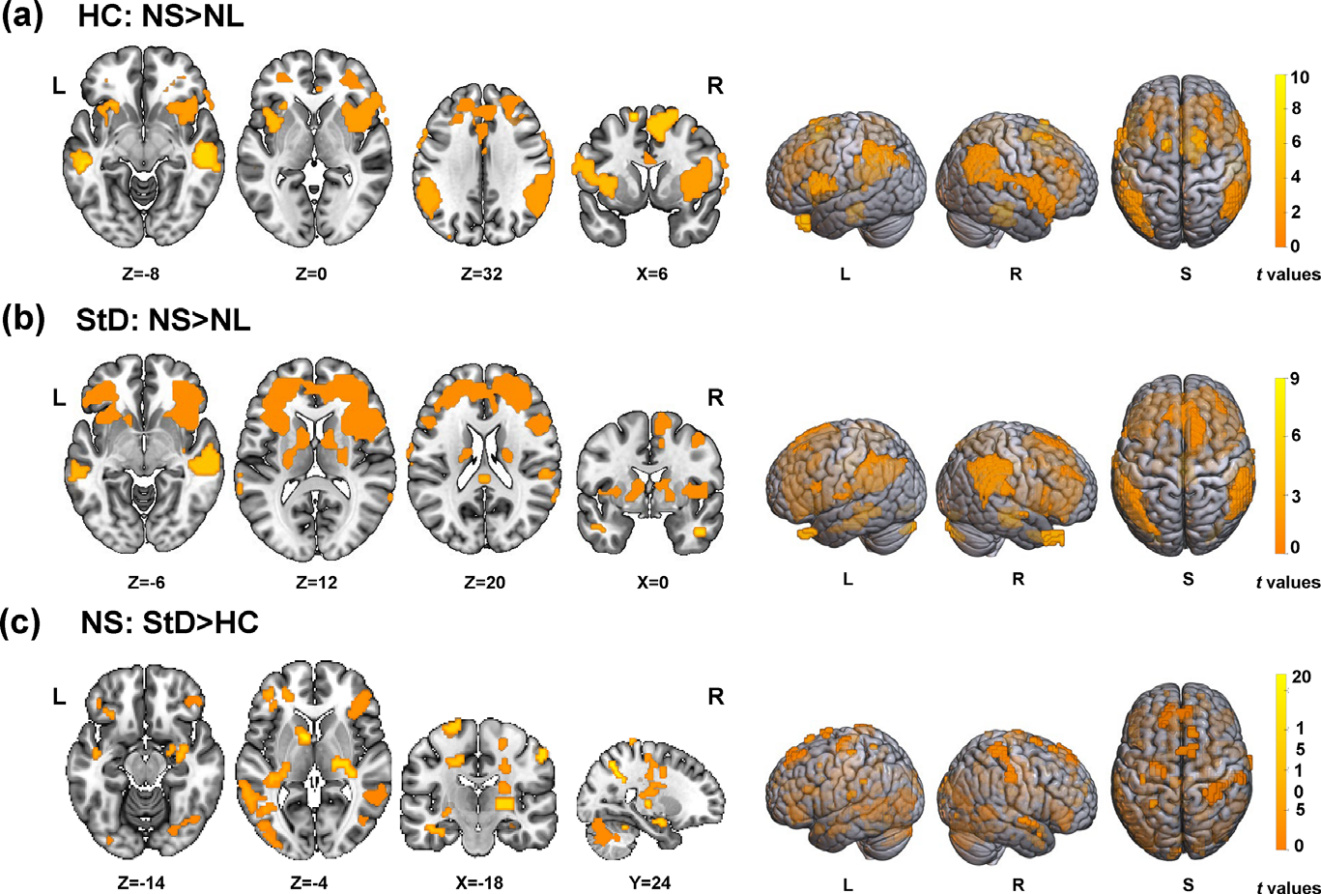
**Impact of the emotion suppression on brain activation. (a)** Images were compared in HC between the NL condition and NS with the paired *t*-tests. **(b)** Images were compared in StD between the NL condition and NS with the paired *t*-tests. **(c)** Images were compared in NS condition between the two groups with the *t*-tests. The results showed that StD was activated in the prefrontal cortex, amygdala, hippocampus and nucleus accumbens when suppressing negative emotion. Increased activation was identified only in the contrast with the threshold of *p* < .001 (uncorrected) and *k* > 30, revealed in MNI coordinates; The color bar represents the *t*-values; dlPFC_L: the left dorsolateral prefrontal cortex; dlPFC_R: the right dorsolateral prefrontal cortex; Amygdala_L: the left amygdala; Amygdala_R: the right amygdala.

Notably, the two groups had different activation patterns. In HC, significant activations (NS-NL maps) were observed in the inferior parietal lobule, superior gyrus, inferior frontal, and middle frontal gyri. Additionally, the bilateral insula showed significant activation, along with the occipital lobe (Figure 3a; Supplementary Table S2).

In StD, significant activations during the NS-NL condition were found in the insula, the inferior frontal gyrus, the superior and middle frontal gyri, the bilateral inferior parietal lobule, the temporal gyrus, the temporal pole, the supramarginal gyrus, the anterior cingulate, and midcingulate cortices. Moreover, activations were seen in subcortical structures like the putamen and caudate, which are associated with motivation and addiction (Figure 3b; Supplementary Table S2).

When comparing the StD participants to the HC (StD-HC), significant differences in brain activation during emotion suppression were observed in the frontal lobe, including the superior and middle frontal gyri and the orbitofrontal cortex. Increased activation was also noted in the hippocampus and amygdala. Additionally, StD participants exhibited heightened activation in the caudate, posterior cingulate cortex, insula, cerebellum, and occipital regions (Figure 3d; Supplementary Table S2).

#### General connectivity patterns and correlation with behavioral outcome

**Figure 4.**
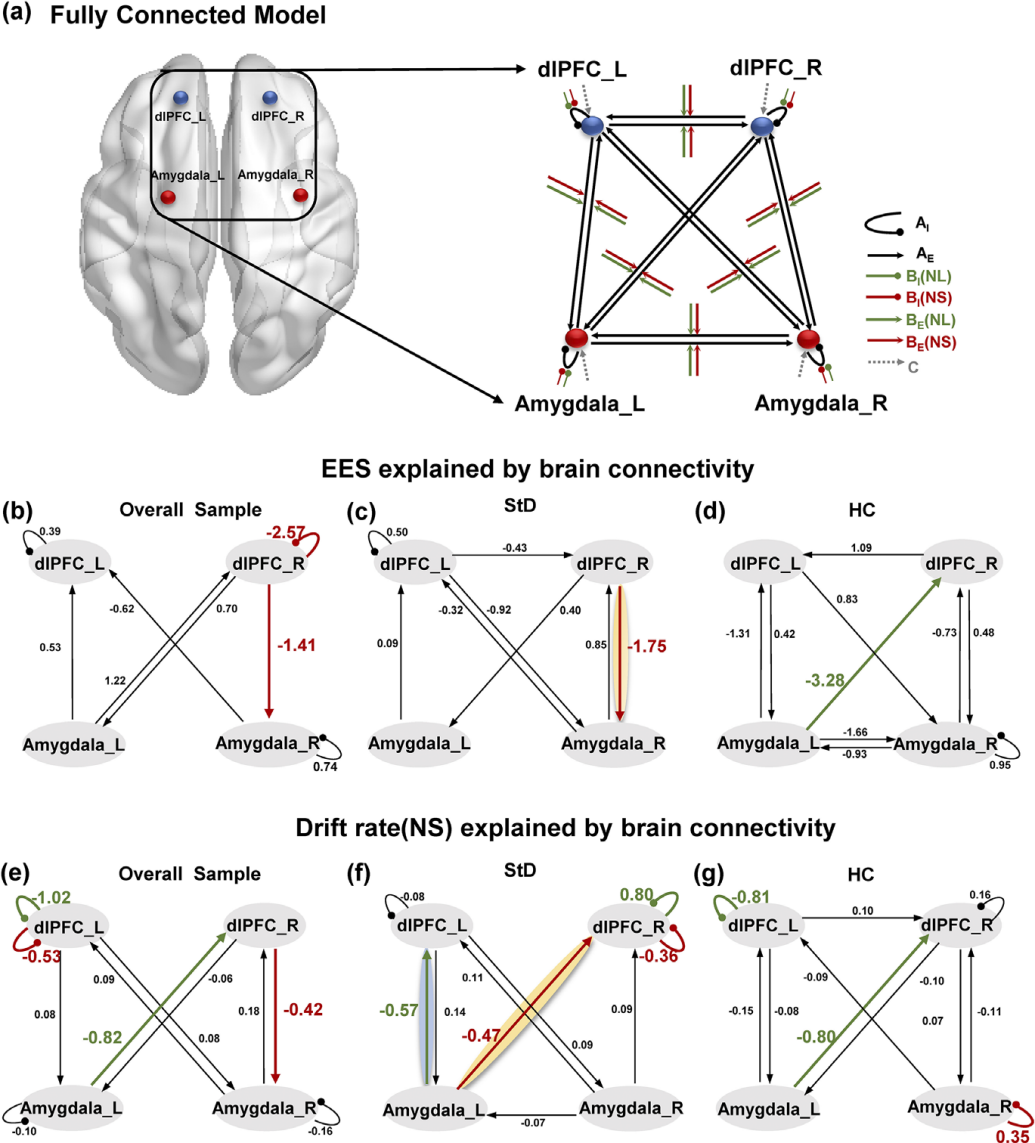
**Impact of the emotion suppression on parameter estimation of group DCM with PEB. (a)** Schematic of modulatory connectivity and driving input. The bilateral dlPFC and amygdala were included as regions in DCM (Montreal Neurological Institute coordinates: left amygdala [-20, 0, -21] and right amygdala [31, 1, -16]) and left dlPFC [-12, 42, 45] and right dlPFC [12, 42, 45]). The dotted lines in black stands for intrinsic selfconnections (AI) and extrinsic between-region connections (AE) without being modulated; The dotted lines in green or red mean that the intrinsic selfconnections and extrinsic between-region connections receive modulatory effects during specific task conditions (BI, BE), and the dotted lines in gray mean that driving input by Task (C matrix). **(b)** The between-participant difference in EES explained by brain connectivity of the overall Sample. The connection of the right dlPFC to the right amygdala could explain the interindividual differences in the EES, and the NS modulated connection from right dlPFC to right amygdala was negatively correction with the EES. **(c)** The EES analysis in StD. The NS modulated right dlPFC to right amygdala connections negatively correlated with EES. **(d)** The EES analysis in HC. The NL modulated connection left amygdala to right dlPFC negatively correlation with EES. **(e)** The between-participant difference in drift rate (NS) explained by brain connectivity of the overall Sample. Similarly, The NS modulated right dlPFC to right amygdala connection negatively correlation with drift rate. Additionally, the drift rate was negatively correlated with the connection from the left dlPFC to the right dlPFC, modulated by NL. **(f)** The drift rate (NS) analysis in StD. The NL modulated left amygdala to left dlPFC connections and NS modulated left amygdala to right dlPFC connections both negatively correlated with drift rate (NS). **(g)** The drift rate (NS) analysis in the HC. Similar to the overall sample, the NL modulated left amygdala to right dlPFC connections negatively correlated with drift rate (NS). dlPFC=the dorsolateral prefrontal cortex; _L=the left hemisphere; _R=the right hemisphere.

The DCM analysis was initially conducted on the entire sample to explore general connectivity patterns. The results indicated that the EES was negatively correlated with the connectivity strength from the right dlPFC to the right amygdala, modulated by NS (−1.41 Hz) (Figure 4b). Similarly, the *δ* showed a negative correlation with the connection from the right dlPFC to the right amygdala, also modulated by NS (−0.42 Hz) (Figure 4e).

We extracted the effective connectivity at the individual level for correlation analysis with EES and *δ* under the NS, and analysis confirmed a negative correlation (*r*_EES_ = −445, *p* = 0.38, Supplementary Figure S3a; *rδ*_(NS)_ = −0.512, *p* = .018, Supplementary Figure S3b). Additionally, the *δ* was negatively correlated with the connection from the left dlPFC to the right dlPFC, modulated by NL (−0.82 Hz) (Figure 4e).

#### Group-specific connectivity patterns and correlation with behavioral outcome

Subsequently, our analysis focused on the HC and StD groups separately to examine whether these patterns persisted within each group and to identify any group-specific differences. In the StD group, the EES analysis demonstrated trends similar to those of the overall sample, with a significant negative correlation between the EES and the right dlPFC → right amygdala connection, modulated by NS (−1.75 Hz) (Figure 4c). In contrast, the HC group showed a negative correlation between EES and the connectivity strength from the left amygdala to the right dlPFC, modulated by NL (−3.28 Hz) (Figure 4d).

Results of the analysis in the HC subgroup paralleled the overall sample trends, showing a significant negative correlation between the *δ* (NS) and the left amygdala to right dlPFC connection, modulated by NL (−0.80 Hz) (Figure 4g). In the StD, the NL-modulated connection from the left amygdala to the left dlPFC was negatively correlated with the *δ* (NS) (−0.57 Hz) (Figure 4f). Additionally, the left amygdala–to–right dlPFC connection modulated by NS also showed a significant negative correlation with the *δ* (NS) (−0.47 Hz) (Figure 4f).

## Discussion

With the aim of identifying the computational behavioral and neural mechanisms of emotional suppression in StD, we conducted laboratory and fMRI studies. We found that, compared to HC, StD participants showed reduced efficacy of EES and a faster *δ* of negative preference. DCM analysis of all samples revealed that the EES of individual differences might be explained by the negative connection from the right dlPFC to the right amygdala modulated by NS. Meanwhile, the *δ* of individual differences may be attributed to a negative connection from the left amygdala to the right dlPFC modulated by NL.

Individuals with StD habitually resort to suppression strategies to manage their negative emotions. However, unlike HC, they do not experience a significant reduction in negative emotions following suppression efforts. This suggests a potential impairment in the emotional regulation capabilities of individuals with StD, which is associated with high emotional inertia (Zheng & Asbury, 2019). Emotional inertia refers to the resistance to change one’s emotional state, implying that a person’s current emotional state is significantly influenced by their prior emotional state (Kuppens, Allen, & Sheeber, 2010). Those with low emotional inertia can more readily bounce back from negative emotional experiences (Longis, Alessandri, Sonnentag, & Kuppens, 2022). The lack of improvement in negative emotions among those with StD after suppression suggests strong emotional inertia, meaning that negative emotional states are more easily maintained or restored. This is further supported by the higher *δ* observed in StD during the NS, indicating a quicker return to negative emotional states after attempts at suppression. These findings align with the concept of emotional inertia, where individuals who experience greater emotional inertia are more likely to sustain or revert to negative emotional states over time (Koval et al., 2015; Kuppens et al., 2012).

The observed propensity of StD to rapidly interpret emotional experiences negatively following suppression attempts may be indicative of an accentuated negativity preference. This preference, which is the tendency to disproportionately attend to negative over positive information (Koval, Brose, et al., 2015), could contribute to the swift resurgence of negative emotional states post-suppression. Such a pattern suggests that individuals with StD may have an increased sensitivity to negative emotional stimuli, further entrenching the negativity bias. Consequently, our findings suggest that a pronounced negativity preference, coupled with greater negative emotional inertia, may exacerbate the challenges of emotion suppression in StD, potentially leading to increased anhedonia.

In light of the behavioral data, StD participants exhibited a level of positive bias comparable to that of HC, and they perceived themselves as effective in suppressing negative emotions. Despite this, their actual suppression outcomes were inferior to those of HC, suggesting a disconnection between intention and outcome in StD. This discrepancy may reflect a motivational conflict where the desire to suppress is undermined by less-successful suppression outcomes. Additionally, the greater activation of the nucleus accumbens observed in StD under NS conditions in the GLM analysis hints at a potentially stronger drive to suppress negative emotions. This could imply that individuals with StD exert more effort or use additional cognitive resources in their attempts to suppress negative emotions. Importantly, although those with StD recruit more dlPFC resources during suppression, their negative emotions are not effectively reduced. DCM further confirmed that a stronger dlPFC → amygdala modulation was paradoxically associated with poorer suppression outcomes. This dissociation between an increased prefrontal effort and unsuccessful behavioral outcomes indicates inefficient top–down regulation of the amygdala. Similar patterns have been observed in recent studies of depression, where patients showed greater dlPFC activation without successful emotion regulation (Gao, Yan, & Yuan, 2022).

The presence of a negativity bias in StD individuals may further compound their struggles with emotional inflexibility and resistance to altering negative emotional states. Such difficulties in emotional regulation can have detrimental effects on mental health and well-being. Consistent with these findings, the drift rates in decision-making processes also mirrored this pattern. Enhanced dlPFC modulation of the amygdala was associated with faster drift rates in decision-making. This suggests that strong dlPFC control over the amygdala may place individuals in a state of heightened cognitive activation during subsequent decision tasks. As a result, this could accelerate the accumulation of evidence, leading to a faster drift rate and potentially affecting the quality and efficiency of decision-making. Importantly, the drift rate in the drift-diffusion model is inherently tied to the specific task and stimulus context, as it reflects the rate of evidence accumulation for emotion-related decisions in our paradigm. A faster drift rate for negative evaluations therefore directly indexes more rapid processing of negative emotional information.

The identification of effective interventions for StD is of importance, given that individuals with this condition often experience persistent depressive symptoms and functional impairment are at heightened risk of developing MDD (Kroenke, 2017; Zhang et al., 2023). Typically, depressive symptoms are linked to the increased use of maladaptive emotion-regulation strategies, including rumination, avoidance, and suppression (Volkaert, Wante, Wiersema, & Braet, 2024; Zhu, Fu, Yan, Yuan, & Yang, 2024). Our findings indicate that StD individuals do not experience an alleviation of negative emotions following attempts at emotional suppression. From a clinical perspective, tailored emotional-regulation training could be developed to assist patients in more effectively managing their negative emotions. Additionally, through DCM analysis, we identified specific patterns of neural connectivity that underlie individual differences in emotional suppression and evaluation, offering potential targets for clinical neuroregulation interventions. Techniques such as transcranial magnetic stimulation and deep brain stimulation have the potential to modulate these neural connectivity patterns and the brain regions implicated in emotional regulation (Croarkin et al., 2021; Noda et al., 2015; Surya, Rosenquist, & McCall, 2016).

At the neural level, our GLM results revealed that StD exhibited heightened activation in both the amygdala and the dlPFC during suppression. This co-activation profile provides important insight into why suppression fails in StD. On the one hand, stronger amygdala responses reflect exaggerated emotional reactivity to negative stimuli, thereby increasing the regulatory demands. On the other hand, increased dlPFC activation suggests that StD recruit additional cognitive resources in an attempt to control these amplified affective responses. For instance, dlPFC activation to sad faces has been shown to scale positively with depression severity, consistent with greater regulatory efforts in both subclinical and clinical depression populations (Chen et al., 2023). At the same time, systematic reviews confirm that antidepressant-free MDD patients show amygdala hyperreactivity to negative stimuli and atypical prefrontal–amygdala connectivity (Tassone et al., 2022).

Critically, this compensatory recruitment appears inefficient: despite greater dlPFC engagement, negative affect was not successfully reduced. The dissociation between neural effort and behavioral outcome implies that, in StD, the prefrontal control system is over-engaged but not effectively coupled to amygdala regulation. In other words, suppression in StD may function as a high-cost but low-yield strategy – mobilizing more cognitive effort while failing to inhibit limbic reactivity. This interpretation is further supported by our DCM findings, which demonstrated that stronger dlPFC-to-amygdala modulation was paradoxically associated with poorer suppression outcomes. Additionally, effective connectivity studies in lifetime MDD report increased inhibitory influence from the amygdala to the dlPFC, suggesting a bottom–up disruption of regulatory circuits (Stolicyn et al., 2023). Meta-analytic evidence further shows that successful emotion downregulation typically depends on effective dlPFC–amygdala coupling (Berboth & Morawetz, 2021); thus, in StD, it is not coupling per se that is absent but rather its dysregulation that underlies suppression failure. Taken together, these findings suggest that the neural circuitry of suppression in StD is marked by both heightened emotional generation and inefficient regulatory control, directly accounting for the observed failure of emotion inhibition.

While this study offers valuable insights into emotional regulation and neural mechanisms in StD, several limitations need consideration. First, the participant pool was primarily university students; future research should include a more diverse demographic to improve generalizability. This study also focused solely on individuals with StD, and future studies should also involve those with MDD and individuals in remission to better understand emotional suppression across the depression spectrum. Second, this was an exploratory study, and the sample size of Sample 2 was determined with reference to prior fMRI research in depression. Nevertheless, for DCM analyses, this sample remains relatively modest, which may restrict the generalizability of our findings. Despite this limitation, the present study represents an initial exploratory effort to characterize the neural mechanisms of emotion suppression in subthreshold depression by combining behavioral modeling with effective connectivity analysis. Future research should extend these findings by employing larger cohorts and longitudinal designs to establish the robustness of the observed effects and to evaluate their potential predictive value for the progression from subthreshold to major depression. Third, a further limitation concerns the interpretation of the drift rate findings. While drift rate is widely recognized as an index of evidence-accumulation efficiency and has been used to capture evaluative biases in affective tasks (Nagrodzki et al., 2025; Ratcliff & McKoon, 2008; Uribe, Bowen, & Meuret, 2025), our paradigm could not fully determine whether the faster drift for negative evaluations in StD reflects a true emotion-specific bias or a more general tendency toward accelerated decision-making under suppression demands. Because the current task was designed specifically for emotional evaluation, it could not provide a non-emotional baseline for comparison. Future studies should therefore incorporate a matched non-emotional decision task to disentangle emotion-specific evaluative biases from domain-general decision tendencies. Fourth, our whole-brain fMRI analyses used a voxel-level uncorrected threshold of *p* < .001 with a cluster extent threshold of *k* > 30, a commonly used exploratory approach in neuroimaging studies (Tsujimoto, Matsuzaki, Yamaya, & Kawashima, 2023; Yeung, 2018). We acknowledge that this does not formally control for family-wise error and may increase susceptibility to false positives. However, our core findings are based on effective connectivity between regions (DCM analysis), and the voxel-wise activations serve primarily to illustrate regional patterns rather than drive the main conclusions. Finally, reliance on self-reported measures and behavioral tasks can introduce biases and fail to capture the complexity of real-world emotional experiences. Moreover, although emotional inertia was discussed as a potential explanatory framework for our findings, it was not directly measured in the present study. Future research would benefit from incorporating ecological momentary assessment, and real-time monitoring could provide a more accurate understanding of daily emotional regulation in individuals with StD.

## Conclusion

This study provides an examination of emotional experiences and brain mechanisms. Behavioral analysis indicated that the negative emotions in StD individuals do not diminish after suppression, and these individuals exhibit quicker judgments of emotional experiences as negative, reflecting both emotional inertia and a heightened negativity bias. Neuroimaging results highlighted that stronger dlPFC → amygdala connections correlate with poorer suppression outcomes and a faster drift rate. This maladaptive regulation may be due to the fact that suppression of StD requires more cognitive effort, investing more top–down effort that does not translate into effective regulation. Taken together, these findings suggest that emotional suppression in StD relies on high-cost but low-yield neural strategies. Overall, these results advance our understanding of the neural circuitry underlying emotional regulation deficits in StD and may inform the development of targeted therapeutic interventions.

## Supporting information

10.1017/S0033291725103097.sm001 Niu et al. supplementary material Niu et al. supplementary material

## Supplementary material

The supplementary material for this article can be found at http://doi.org/10.1017/S0033291725103097.
