# Supplementary material for: The role of altered decision dynamics and dorsolateral prefrontal cortex to amygdala causal circuitry in the aberrant efficacy of emotion suppression in subthreshold depression
Source: Psychol Med. 2026 Jan 30;56:e34. doi: 10.1017/S0033291725103097 (PMC12902176; doi:10.1017/S0033291725103097)
Supplement: Niu et al. supplementary material [file S0033291725103097sup001.docx]

Supplementary

**The Role of Altered Decision Dynamics and Dorsolateral Prefrontal Cortex to Amygdala Causal Circuitry in the Aberrant Efficacy of Emotion Suppression in Subthreshold Depression**

**Contents**

[Methods 2](#_Toc184125882)

[Participant recruitment and selection process: a two-stage method 2](#_Toc184125883)

[Sample1 2](#_Toc184125884)

[Sample2 2](#_Toc184125885)

[Experimental material 3](#_Toc184125886)

[Imaging data acquisition and preprocessing 3](#_Toc184125887)

[General linear model analysis for fMRI data 4](#_Toc184125888)

[Results 4](#_Toc184125889)

[The ANOVA results for the decision threshold, initial bias and non-decision in Sample 1 4](#_Toc184125890)

[Poorer Emotion Suppression Effects of StD in Sample 2 4](#_Toc184125891)

[Faster Drift Rates for Negative Options of StD in Sample 2 5](#_Toc184125892)

[The ANOVA results for the decision threshold, initial bias and non-decision in Sample 2 5](#_Toc184125893)

[References 5](#_Toc184125894)

[Table S1. Experimental stimulus valence and arousal. 6](#_Toc184125895)

[Table S2. Activated regions were revealed by the general linear model analysis. 6](#_Toc184125896)

# Methods

## Participant recruitment and selection process: a two-stage method

### Sample1

We adopted a two-stage procedure consisting of an initial screening and diagnostic confirmation. In the first screening stage, students from the Universities were randomly invited to complete both the Beck Depression Inventory-II (BDI-II) and the Center for Epidemiological Studies Depression Scale (CES-D). We used both the BDI-II and CES-D to identify potential StD candidates based on established cutoffs. The rationale for using two scales in combination was to improve the reliability of classification, as single-scale screening may be influenced by response bias or instrument-specific measurement variance. Students majoring in psychology and psychiatry were not on the list. A total of 319 young adults finished completing the scales.

We first excluded those participants who did not finish the questionnaires or did not give their correct contact information. Then, since CES-D included 4 reverse-scored items, it was used to further exclude participants who showed the same reaction trend between the reverse-scored items and the forward-scored items (Mei et al., 2020). By previous studies (Gotlib et al., 1988; Li et al., 2015), a BDI score of 6 and below indicated that an individual was not depressed while a score of 14 and above indicated a minor depression. And in CES-D, a score of 16 was the critical point of depression (Radloff & L., 1977). Participants who scored above the cutoffs on both scales were considered potential StD and were invited to the second stage.

In the second diagnostic confirmation stage, participants who scored 14 or above in BDI and scored 16 or above in CES-D were invited to have an International Neuropsychiatric Interview (MINI) conducted by two trained postgraduate students in the Department of Psychology. The MINI was used to systematically exclude diagnosable psychiatric disorders (with emphasis on current or past MDD) and to verify the presence of at least one core depressive symptom (depressed mood or anhedonia) at a subthreshold level (i.e., symptom presence without meeting full MDD criteria). Participants meeting any DSM-defined psychiatric diagnosis on the MINI were excluded. According to the interview results, the following exclusion criteria were used to select participants: (a) fulfill the DSM-IV diagnostic criteria for MDD; (b) a self-reported history of neurological or psychiatric disorders; (c) a history of taking any psychotropic drugs. All participants had normal or corrected vision.

Finally, 55 subjects who passed the M.I.N.I. and exclusion criteria were assigned to the StD group (M_age_=21.60, M_SD_=2.32, ages 18–27, female=30, male=25), whereas 60 subjects with a BDI score of 6 or below and a CES-D score of 16 or below were directly assigned to the HC group (M_age_=20.71, M_SD_=2.61, ages 18–32, female=35, male=25) (Fig 1a).

### Sample2

The recruitment and selection process for Sample 2 was the same as for Sample 1. In addition to meeting the general inclusion criteria, all participants had to comply with the following magnetic resonance imaging (MRI) scan inclusion criteria: (a) No cardiac pacemakers, electronic cochleas, tattoos, prostheses, or any metallic implants in the body, including aneurysm clips, metal fragments, stents, or other metallic devices that could interfere with the MRI; (b) No history of claustrophobia or severe anxiety that could be exacerbated by the MRI environment; (c) No significant neurological or psychiatric disorders that could affect MRI results; (d) No prior head injuries or surgeries involving metallic implants; (e) Ability to remain still for the duration of the MRI scan; (f) No pregnancy, due to the potential risks associated with MRI exposure; (g) Normal or corrected-to-normal vision and hearing, to ensure compliance with task instructions during the MRI scan.

Finally, 25 subjects were assigned to the StD group, whereas 25 subjects were assigned to the HC group. Four participants withdrew from the experiment due to the remote location of the experiment site and scheduling conflicts. Two participants requested to suspend the experiment due to excessive noise during the stimulus presentation and scanning, which caused anxiety and physical discomfort. One participant was not allowed to proceed with the scan because they had gold-colored nail polish. Therefore, Sample 2 involved 23 subjects in the StD group (M_age_=22.17, M_SD_=2.37, ages 18–27; female=5, male=18) and 20 subjects in the HC group (M_age_=22.29, M_SD_=3.50, 18–32 years, female=7, male=13) (Fig 1a).

## Experimental material

Pictures were chosen from the International Affective Picture System(IAPS) (Bradley & Lang, 1994). We first selected 70 negative pictures (valence: 2.74±0.55, arousal:5.51±0.93) and 40 neutral pictures (valence:4.99±0.39, arousal:3.58±1.11) with no repetition, and then we invited 9 students to re-rate the valence (0: Extremely unpleasant, 100: Extremely pleasant) and arousal (0: Extremely calm, 100: Extremely excited) of all pictures on a 0-100 scale. We selected 60 negative pictures with the lower valence score and 30 neutral pictures with the middle valence score as formal experimental materials in Sample 1.

For the experimental materials in Sample 2, we randomly selected 30 negative and 15 neutral pictures from those used in Sample 1, forming a formal experimental set of 45 trials (Table S1).

## Drift Diffusion Modeling

### Data Preparation

All reaction times (RTs) were first converted into seconds. Participants’ ratings were then recoded into a binary decision variable: ratings 1–4 were coded as 0, corresponding to the lower boundary (negative evaluation), and ratings 5–8 were coded as 1, corresponding to the upper boundary (positive evaluation). Finally, trials with extreme or missing responses were excluded, specifically those with RT < 0.15 s, RT > 10 s, or without a recorded response.

### Mathematical Formulation

The decision-making process for emotional evaluation was modeled using a standard drift diffusion framework. The model assumes that evidence accumulates over time until a decision threshold is reached. The accumulation of evidence X_t_ was modeled as a one-dimensional Wiener process with drift:

$dX_{t}=v dt+s dW_{t}, X_{0}=z, boundaries(0,a)$,

where $v=\delta$ is the drift rate, $a=\alpha$ is the boundary separation (decision threshold), $z=\beta$ is the starting point (initial bias), and $s$ is the diffusion constant (fixed to 1 for identifiability). $dW_{t}$ increment of a Wiener process (Gaussian noise). The non-decision time $\tau$ accounts for perceptual and motor latencies, such that the observed reaction time (RT) is given by:

$$RT=T_{decision+\tau}$$

where $T_{decision}$is the first-passage time (FPT).

### Parameter definitions and priors

Drift rate (*δ*): The drift rate represents both the speed and direction of evidence accumulation, with positive values reflecting a tendency toward positive ratings and negative values toward negative ratings. We placed an unconstrained prior $v$∼N(0,1) on the drift rates. This weakly informative prior reflects the expectation that most drift rates are centered around zero but can vary in either direction.

Decision threshold (*α*): The decision threshold reflects response caution or the amount of evidence required before committing to a choice. Larger *α* values correspond to slower but more consistent decisions. For each instruction, we placed a uniform prior $a$∼Uniform (0.1,10), representing a flat prior that covers a broad and theoretically plausible range based on prior empirical findings.

Starting point bias (*β*): The starting point captures initial predisposition toward one response before evidence accumulation begins. We placed a uniform prior $z_{r}$∼Uniform(0,1) on the relative starting point in the aversive and neutral conditions, ensuring the full plausible range of initial bias was covered. For reappraisal trials, the starting point bias was constrained to lie between the corresponding estimates for neutral and aversive conditions, consistent with theoretical expectations about reappraisal effects.

Non-decision time (*τ*): The non-decision time accounts for perceptual encoding and motor execution processes. For each individual, *τ* was fixed across instruction and stimulus types, reflecting its non-cognitive nature. We placed a uniform prior $\tau$∼Uniform (0, RT_min_), where RT_min_ ​ denotes the shortest valid reaction time for that individual, ensuring that *τ* does not exceed the minimum possible RT.

### Model Specification

The model included the following parameters: boundary separation ($a$), drift rate ($v$), starting point ($z$), and non-decision time ($\tau$). Condition-dependent effects, such as variations in drift rate ($v$) across instructions or stimulus types, were specified using the *depends_on* argument or regression formulas via HDDMRegressor.

### Model Estimation and fitting (Docker HDDM)

## The decision-making process for emotional evaluation was modeled using a standard drift diffusion framework, implemented in the HDDM package (<https://hub.docker.com/r/hcp4715/hddm/tags>)(Wanke et al., 2022). We adopted a hierarchical Bayesian estimation approach using Markov Chain Monte Carlo (MCMC) sampling. A recommended initialization strategy was employed: four parallel chains, each with 10000 iterations, of which the first 2000 iterations were discarded as burn in, and every 2nd sample was retained (thinning = 2). This resulted in approximately 4000 posterior samples per chain. These values serve as an example and should be adjusted according to model complexity, with more iterations required for more complex models to ensure stable convergence.

## Imaging data acquisition and preprocessing

All imaging data were collected on a 3T Siemens Trio Tim scanner with a 64-channel phased-array head coil in the Brain Imaging Center of South China Normal University (SCNU). Task-based functional imaging was performed using an EPI gradient-recalled echo-planar imaging sequence to collect blood oxygen level-dependent (BOLD) signals in the brain task with the following parameters: TR=1500 milliseconds; TE=31 milliseconds; slice thickness=2.4mm; flip angle=70 degrees; FOV=88mm×88mm 60mm; voxel-size=2.398mm×2.398mm×2.4mm) and acquisition time of 15 minutes and 45 seconds. In addition, high-resolution brain structural images were acquired using a T1-weighted 3D MP-RAGE sequence with the following parameters: TR=1800 milliseconds; TE=2.07 milliseconds; slice thickness=0.8mm; flip angle=9 degrees; FOV=320mm×320mm×208mm; voxel-size =0.8mm×0.8mm×0.8mm).

All images were converted from DICOM format to NII format by MRIConvert, and the subsequent image processing was carried out by the MatlabR2018b platform. Task-functional fMRI data were preprocessed using Statistical Parametric Mapping software (SPM12; http://www.fil.ion.ucl.ac.uk/spm/software/spm12/). The specific steps of preprocessing were as follows: (1) realign: The automatic image matching method was used to correct the head motion generated by each participant, excluding participants with head motion parameters more than 2mm or 2°; (2) normalize: performing spatial normalization to the Montreal Neurological Institute (MNI) space by applying new segments to the structural images (resampling voxel size=3mm×3mm×3mm); (3) smooth: performing spatial smoothing using an isotropic Gaussian kernel of 6mm full width at half maximum (FWHM).

## General linear model analysis for fMRI data

For each participant, we first conducted a first-level general linear model (GLM) analysis to calculate the activation maps for the NS condition relative to the NL condition (NS-NL). Subsequently, we performed a second-level analysis. For within-group analyses, we conducted one-sample t-tests on the NS-NL maps for both the HC and StD groups separately, identifying brain regions significantly activated under the NS condition within each group. Next, to further identify the differences between the two groups, we conducted independent two-sample t-tests on the NS-NL maps. The results revealed significant differences in brain activation between the StD group and the HC group during emotion suppression. Activations reported survived an uncorrected voxel-level intensity threshold of *p*< .05 with a minimum cluster size of k>30 voxels.

## Dynamic Causal Modeling (DCM) and Group-Level Analysis

Dynamic Causal Modeling (DCM) was used to infer the directed causal interactions among the predefined regions of interest (ROIs). DCM models neuronal dynamics as a bilinear state-space system, expressed as:

$$x\left( t \right)=(A+\sum_{j} u_{j}B_{j})x(t)Cu(t)$$

where $x(t)$ denotes neuronal states of the ROIs, $u(t)$ represents experimental inputs, matrix A encodes fixed (intrinsic) connectivity that is present across all conditions, matrix B represents condition-specific modulatory influences on those connections, and matrix C specifies how external inputs drive activity within the network. In this framework, the observed BOLD signal is treated as a nonlinear hemodynamic transformation of hidden neuronal states, allowing for causal inference about neural interactions rather than simple correlations.

### ROI Definition and Time Series Extraction

ROIs were selected based on group level GLM peaks in the bilateral dlPFC and amygdala, regions consistently implicated in emotion regulation. For each participant, an 8mm radius sphere centered on the subject-specific activation peak was created. The principal eigenvariate of the BOLD time series was extracted using SPM’s eigenvariate tool, adjusted for confounds (motion, feedback-related events, and session means) via *F* contrasts. This procedure isolates task relevant neural fluctuations while reducing noise and individual variability.

### Model Specification and Priors

We adopted a fully connected model structure, where all ROIs were assumed to be bidirectionally connected (matrix A). Experimental task inputs were assigned to matrix C to drive dlPFC activity, consistent with its role in initiating regulatory control. Matrix B captured condition-specific modulation (e.g., EES, *δ*(NS)), representing how task demands altered effective connectivity. Priors followed standard SPM12 defaults (connection strengths centered at 0 Hz with a prior variance of 0.04), ensuring conservative estimates. By estimating deviations from these priors, DCM infers how strongly task manipulations influence directed connections.

### Model Estimation (Variational Laplace)

Each subject’s DCM was inverted using Variational Laplace, a Bayesian scheme that optimizes the posterior density over parameters by maximizing variational free energy. This provides both point estimates and uncertainty bounds (posterior covariance) for each connection. Convergence was checked by inspecting the free energy and ensuring stable posterior estimates across iterations.

### Group-Level Inference (Parametric Empirical Bayes, PEB)

To integrate individual models, we employed a Parametric Empirical Bayes (PEB) framework. PEB is a hierarchical Bayesian approach in which first-level (subject-specific) DCM parameters are treated as observations, and a second-level model estimates group mean connectivity and covariate effects. This framework naturally incorporates subject-level uncertainty into group inference. The design matrix included five regressors: group mean connectivity, EES, *δ*(NS), CES-D, and BDI scores. All regressors were mean-centered and z-scored prior to entry. Posterior probabilities quantified the strength of evidence for each parameter, with >95% indicating strong evidence for a connection’s presence.

### Model Optimization (Bayesian Model Reduction, BMR)

After PEB estimation, Bayesian Model Reduction (BMR) was applied to prune parameters that did not contribute to model evidence. BMR is an iterative “greedy search” procedure that compares nested models by switching parameters on and off, retaining only those that improve log-evidence. This ensures a parsimonious final model, avoiding overfitting while maximizing explanatory power.

### Model Validation and Robustness Checks

Predictive validity was evaluated using leave-one-out cross-validation (LOOCV), in which the group-level PEB model was refitted excluding one participant, and the estimated parameters were used to predict that participant’s behavioral performance. Prediction accuracy was quantified by Pearson correlations between predicted and observed behavioral measures. This procedure confirmed that task-related effective connectivity, particularly modulatory effects in matrix B, reliably predicted individual differences in behavioral outcomes.

# Results

## The ANOVA results for the decision threshold, initial bias and non-decision in Sample 1

The two-way ANOVA for the decision threshold indicated a significant main effect of condition (*F*_(2,113)_ =45.214, *p*<.001), but no significant main effect of group (*F*_(1,113)_ =2.503, *p*=.115) and no significant interaction effect between group and condition (*F*_(2,113)_ =1.258, *p*=.286). Given the significant main effect of condition in decision threshold, for HC, the post-hoc test revealed significant differences between Neutral vs. NL (Mean Difference =-0.775, *p*<.001), Neutral vs. NS (Mean Difference=-0.926, *p*<.001). Similarly, StD showed significant differences between Neutral vs. NL (Mean Difference=-1.060, *p*<.001) and Neutral vs. NS (Mean Difference=-1.309, *p*<.001) (Fig.S1).

The two-way ANOVA for initial bias and non-decision time showed no significant main effects of group (*τ*: *F*_(1,113)_ =0.524, *p*=.470; *τ*: *F*_(1,113)_ =1.583, *p*=.209) or condition (*β*: *F*_(2,113)_ =1.504, *p*=.224; *τ*: *F*_(1,113)_ =0.222, *p*=.801), and no significant interaction effect (*β*: *F*_(2,113)_ =0.265, *p*=.767; *τ*: *F*_(1,113)_ =0.018, *p*=.982).

## Poorer Emotion Suppression Effects of StD in Sample 2

In Sample 2, we found consistent results in which the negative stimuli successfully evoked almost the same degree of negative emotion in both groups (HC: *t*_19_=11.81, *p*<.001; StD: *t*_22_=9.42, *p*<.001; *t*_41_=-0.32, *p*=.752; Fig.1f), the suppression effect in the StD group was significantly worse than in the HC group (M_HC_=0.51, M_StD_=-0.06, *t*_41_=2.36, *p*=.023; Fig.1g). Similarly, participants in both groups perceived themselves as successful in inhibiting their emotions (negative run: M_HC_=5.49, M_StD_=5.23, *t*_41_=1.02, *p*=.314; Fig.1h).

## Faster Drift Rates for Negative Options of StD in Sample 2

The two-way ANOVA for drift rate revealed significant main effects of both groups (*F*_(1,41)_ =10.526, *p*<.001) and condition (*F*_(2,41)_ =125.435, *p*=.002). The interaction effect between group and condition was not significant (*F*_(2,41)_ =0.251, *p*=.778). Given the significant main effect of the condition, post-hoc analyses using Tukey's HSD test were performed to further explore the differences between conditions within each group. For HC, the results revealed significant differences between Neutral vs. NL (Mean Difference=1.362, *p*<.001), and Neutral vs. NS (Mean Difference=1.355, *p*<.001). Similarly, for StD, the Tukey HSD test showed significant differences between Neutral vs. NL (Mean Differenc =1.438, *p*<.001) and Neutral vs. NS (Mean Difference=1.502, *p*<.001). More importantly, given the significant main effects found in the Group, the independent sample t-test found significant differences between the two groups under the NS condition (M_HC_=-1.01, M_StD_=-1.36, *t*_41_=2.61, *p*=.013; Fig.3d).

The correlation analysis results showed that EES was positively associated with drift rate (*r*=0.357, *p*=.019; Fig.2e) and negative correlation with decision threshold in the NS condition (*r*=-0.339, *p*=.026; Fig.S2b).

## The ANOVA results for the decision threshold, initial bias and non-decision in Sample 2

The two-way ANOVA for decision threshold indicated a significant main effect of condition (*F*_(2,41)_ =17.704, *p*<.001), but no significant main effect of group (*F*_(1,41)_ =1.310, *p*=.255) and no significant interaction effect between group and condition (*F*_(2,41)_ =0.686, *p*=.506). Given the significant main effect of Condition in decision threshold, For HC, the Tukey HSD test revealed significant differences between Neutral vs. NL (Mean Difference= -1.189, *p*=.010), Neutral vs. NS (Mean Difference=-1.197, *p*=.009). Similarly, StD showed significant differences between Neutral vs. NL (Mean Difference=-1.594, *p*=.001) and Neutral vs. NS (Mean Difference=-1.857, *p*<.001).

The two-way ANOVA for initial bias and non-decision time showed no significant main effects of group (*β*: *F*_(1,41)_ =0.063, *p*=.802; *τ*: *F*_(1,41)_ =0.455, *p*=.501) or condition (*β*: *F*_(2,41)_ =2.165, *p*=.119; *τ*: *F*_(1,41)_ =0.009, *p*=.991), and no significant interaction effect (*β*: *F*_(2,41)_ =0.770, *p*=.465; *τ*: *F*_(1,41)_ =0.015, *p*=.985).

# References

Bradley, M. M., & Lang, P. J. (1994). Measuring emotion: the Self-Assessment Manikin and the Semantic Differential. *Journal of Behavior Therapy and Experimental Psychiatry*, *25*(1), 49-59. <https://pubmed.ncbi.nlm.nih.gov/7962581>

Gotlib, I. H., Mclachlan, A. L., & Katz, A. N. (1988). Biases in Visual Attention in Depressed and Nondepressed Individuals. *Cognition and Emotion*, *2*(3), 185-200.

Li, H., Wei, D., Sun, J., Chen, Q., Zhang, Q., & Qiu, J. (2015). Brain structural alterations associated with young women with subthreshold depression. *Scientific Reports*, *5*, 9707. <https://doi.org/10.1038/srep09707>

Mei, G., Li, Y., Chen, S., Cen, M., & Bao, M. (2020). Lower recognition thresholds for sad facial expressions in subthreshold depression: a longitudinal study. *Psychiatry Research*, *294*, 113499. <https://doi.org/10.1016/j.psychres.2020.113499>

Radloff, & L., S. (1977). The CES-D Scale A Self-Report Depression Scale for Research in the General Population. *Applied Psychological Measurement*, *1*(3), 385-401.

Wanke, P., Geng, H., Zhang, L., Fengler, A., Frank, M., Zhang, R.-Y., & Chuan-Peng, H. (2022). *dockerHDDM: A user-friendly environment for Bayesian Hierarchical Drift-Diffusion Modeling*. <https://doi.org/10.31234/osf.io/6uzga>

##

## Table S1. Experimental stimulus valence and arousal.

| IAPS number | Valence | Arousal | IAPS number | Valence | Arousal | IAPS number | Valence | Arousal |
| --- | --- | --- | --- | --- | --- | --- | --- | --- |
| Negative | | | Negative | | | Neutral | | |
| 2490.jpg | 42.78 | 50.89 | 2053.jpg^2^ | 29.11 | 54.00 | 2038.jpg | 58.67 | 45.78 |
| 2110.jpg^2^ | 41.89 | 50.22 | 9592.jpg | 28.67 | 57.00 | 2190.jpg | 51.56 | 39.33 |
| 2141.jpg | 41.89 | 50.33 | 9180.jpg^2^ | 28.56 | 45.67 | 2191.jpg^2^ | 59.44 | 47.89 |
| 9427.jpg | 40.44 | 58.00 | 9250.jpg^2^ | 28.56 | 51.78 | 2210.jpg^2^ | 44.44 | 44.78 |
| 3300.jpg | 40.11 | 44.11 | 1301.jpg^2^ | 28.22 | 47.11 | 2280.jpg | 51.44 | 38.33 |
| 9046.jpg^2^ | 39.89 | 41.56 | 2205.jpg | 28.22 | 52.78 | 2393.jpg^2^ | 54.89 | 41.56 |
| 2900.jpg^2^ | 39.56 | 48.00 | 9181.jpg^2^ | 27.89 | 46.78 | 3210.jpg^2^ | 47.67 | 35.67 |
| 9495.jpg | 39.00 | 50.56 | 9611.jpg | 27.89 | 57.67 | 5471.jpg^2^ | 59.33 | 41.56 |
| 3280.jpg | 38.11 | 51.11 | 6250.jpg^2^ | 27.78 | 51.11 | 5740.jpg | 57.67 | 44.89 |
| 2278.jpg | 37.56 | 49.00 | 9050.jpg^2^ | 27.44 | 60.00 | 6150.jpg^2^ | 52.11 | 38.56 |
| 2799.jpg^2^ | 36.89 | 45.11 | 6243.jpg | 26.22 | 44.22 | 6910.jpg | 48.11 | 45.22 |
| 4621.jpg | 36.78 | 66.78 | 1201.jpg | 26.11 | 61.44 | 7000.jpg^2^ | 51.00 | 30.22 |
| 9520.jpg^2^ | 36.22 | 42.00 | 7360.jpg^2^ | 25.67 | 55.67 | 7002.jpg | 48.33 | 30.67 |
| 9421.jpg | 35.89 | 51.78 | 6821.jpg | 25.44 | 67.33 | 7009.jpg^2^ | 51.67 | 30.89 |
| 9530.jpg | 35.11 | 48.56 | 9921.jpg | 25.11 | 51.44 | 7025.jpg | 52.44 | 38.89 |
| 9430.jpg | 35.11 | 47.44 | 6530.jpg^2^ | 24.56 | 70.89 | 7041.jpg^2^ | 50.11 | 31.89 |
| 2120.jpg | 34.67 | 54.44 | 6312.jpg^2^ | 24.33 | 57.78 | 7052.jpg | 48.11 | 36.33 |
| 9903.jpg^2^ | 34.67 | 45.33 | 6838.jpg | 24.33 | 63.00 | 7057.jpg^2^ | 55.22 | 40.00 |
| 9342.jpg^2^ | 34.00 | 49.22 | 3350.jpg^2^ | 23.33 | 46.00 | 7059.jpg^2^ | 49.44 | 31.89 |
| 2276.jpg | 33.56 | 52.33 | 2730.jpg^2^ | 21.56 | 73.33 | 7090.jpg | 49.00 | 30.67 |
| 3230.jpg | 33.44 | 45.78 | 9252.jpg^2^ | 20.89 | 60.78 | 7100.jpg | 49.67 | 28.00 |
| 9290.jpg^2^ | 33.33 | 47.56 | 9635.jpg | 20.89 | 60.56 | 7130.jpg | 60.00 | 32.56 |
| 9041.jpg | 33.00 | 48.78 | 7380.jpg^2^ | 18.00 | 66.89 | 7205.jpg^2^ | 58.00 | 36.89 |
| 9622.jpg | 32.56 | 55.89 | 2095.jpg^2^ | 17.89 | 60.33 | 7217.jpg | 48.00 | 32.78 |
| 9560.jpg^2^ | 32.44 | 46.67 | 6550.jpg | 17.89 | 65.11 | 7491.jpg | 55.11 | 35.22 |
| 9429.jpg | 31.78 | 52.78 | 6560.jpg | 17.56 | 62.33 | 7504.jpg^2^ | 55.00 | 44.67 |
| 2703.jpg^2^ | 31.22 | 53.22 | 1932.jpg^2^ | 17.22 | 57.78 | 7560.jpg^2^ | 54.22 | 31.33 |
| 9600.jpg^2^ | 30.89 | 54.00 | 9254.jpg^2^ | 15.56 | 69.00 | 7640.jpg | 48.78 | 55.33 |
| 9341.jpg^2^ | 30.44 | 43.00 | 1274.jpg | 12.89 | 68.11 | 8211.jpg^2^ | 53.44 | 45.89 |
| 9102.jpg | 30.00 | 57.33 | 9253.jpg^2^ | 12.56 | 76.33 | 9700.jpg | 51.56 | 35.33 |

Note: All pictures used in Sample, .jpg^2^ represent randomly selected pictures used

## Table S2. Activated regions were revealed by the general linear model analysis.

|  | Region | Cluster | Peak MNI coordinates | | | *t*-score |
| --- | --- | --- | --- | --- | --- | --- |
|  |  |  | x | y | z |  |
| HC: NS-NL | Parietal_Inf_R | 214 | 57 | -57 | 42 | 4.42 |
|  | Parietal_Inf_L | 627 | -57 | -54 | 39 | 3.70 |
|  | SupraMarginal_R | 302 | 54 | -39 | 39 | 3.11 |
|  | SupraMarginal_L | 118 | -60 | -45 | 33 | 3.37 |
|  | Frontal_Inf_Oper_R | 204 | 45 | 18 | 9 | 4.06 |
|  | Frontal_Inf_Oper_L | 422 | -57 | 12 | 21 | 3.66 |
|  | Frontal_Sup_L | 44 | -15 | 12 | 69 | 2.08 |
|  | Frontal_Sup_R | 104 | 15 | 12 | 57 | 2.40 |
|  | Frontal_Mid_R | 544 | 33 | 42 | 21 | 3.87 |
|  | Frontal_Mid_L | 375 | -36 | 39 | 21 | 3.26 |
|  | Insula_L | 141 | -36 | 15 | 9 | 3.11 |
|  | Insula_R | 197 | 36 | 12 | 9 | 3.51 |
|  | Temporal_Mid_R | 207 | 57 | -24 | -12 | 3.65 |
|  | Temporal_Mid_L | 87 | -57 | -24 | -12 | 2.90 |
|  | Temporal_Mid_R | 122 | 60 | -21 | -12 | 3.42 |
|  | Cingulum_Ant_L | 91 | -7 | 36 | 14 | 2.11 |
|  | Cingulum_Ant_R | 170 | 10 | 42 | 28 | 2.46 |
|  | Cingulum_Mid_R | 329 | 12 | 12 | 45 | 3.20 |
| StD: NS-NL | Insula_R | 237 | 33 | 24 | 6 | 6.11 |
|  | Insula_L | 199 | -33 | 6 | 6 | 2.79 |
|  | Frontal_Inf_Tri_R | 277 | 48 | 36 | 0 | 6.04 |
|  | Frontal_Inf_Tri_L | 318 | -39 | 36 | 0 | 4.50 |
|  | Frontal_Sup_R | 374 | 24 | 48 | 21 | 5.93 |
|  | Frontal_Sup_L | 162 | -16 | 45 | 33 | 3.08 |
|  | Frontal_Sup_Medial_L | 340 | -6 | 45 | 33 | 3.81 |
|  | Frontal_Sup_Medial_R | 327 | 10 | 45 | 41 | 3.74 |
|  | Frontal_Mid_R | 712 | 30 | 45 | 12 | 4.93 |
|  | Frontal_Mid_L | 413 | -30 | 45 | 12 | 3.92 |
|  | Cingulum_Ant_R | 203 | 6 | 45 | 15 | 3.58 |
|  | Cingulum_Mid_R | 86 | 6 | -24 | 36 | 1.98 |
|  | Parietal_Inf_L | 298 | -54 | -45 | 45 | 4.88 |
|  | Parietal_Inf_R | 175 | 55 | -39 | 47 | 2.95 |
|  | SupraMarginal_L | 155 | -54 | -48 | 32 | 3.42 |
|  | SupraMarginal_R | 376 | 54 | -45 | 33 | 6.24 |
|  | Temporal_Mid_L | 294 | -60 | -21 | -15 | 3.35 |
|  | Temporal_Mid_R | 143 | 54 | -30 | -8 | 2.87 |
|  | Temporal_Pole_Mid_R | 112 | 48 | 15 | -39 | 4.26 |
|  | Putamen_L | 94 | -27 | 5 | 9 | 2.53 |
|  | Putamen_R | 113 | 33 | 5 | 9 | 2.45 |
|  | Caudate_L | 57 | -9 | 6 | 9 | 4.22 |
|  | Caudate_R | 44 | 12 | 5 | 10 | 2.59 |
|  | Pallidum_L | 39 | -12 | 5 | -1 | 2.08 |
|  | Pallidum_R | 39 | 16 | 5 | 0 | 2.42 |
| StD-HC | Frontal_Sup_Medial_L | 164 | -9 | 42 | 42 | 1.89 |
|  | Frontal_Sup_Medial_R | 32 | 12 | 42 | 45 | 1.72 |
|  | Hippocampus_L | 19 | -30 | -6 | -24 | 1.86 |
|  | Hippocampus_R | 30 | 24 | -8 | -18 | 1.97 |
|  | Amygdala_L | 15 | -20 | 0 | -21 | 2.12 |
|  | Amygdala_R | 22 | 24 | -3 | -21 | 2.07 |
|  | Caudate_L | 32 | -12 | 9 | 6 | 1.76 |
|  | Cingulum_Post_L | 42 | -3 | -41 | 28 | 1.69 |
|  | Occipital_Inf_L | 24 | -33 | -87 | -9 | 2.23 |
|  | Occipital_Mid_L | 189 | -33 | -87 | 12 | 3.10 |
|  | Temporal_Mid_R | 193 | 54 | -54 | 4 | 2.29 |
|  | Occipital_Mid_R | 110 | 42 | -75 | 12 | 3.09 |
|  | Cerebelum_Crus2_R | 284 | 30 | -72 | -30 | 1.96 |
|  | Cerebelum_Crus2_L | 131 | -15 | -87 | -32 | 2.37 |
|  | Postcentral_R | 64 | 39 | -30 | 63 | 1.77 |


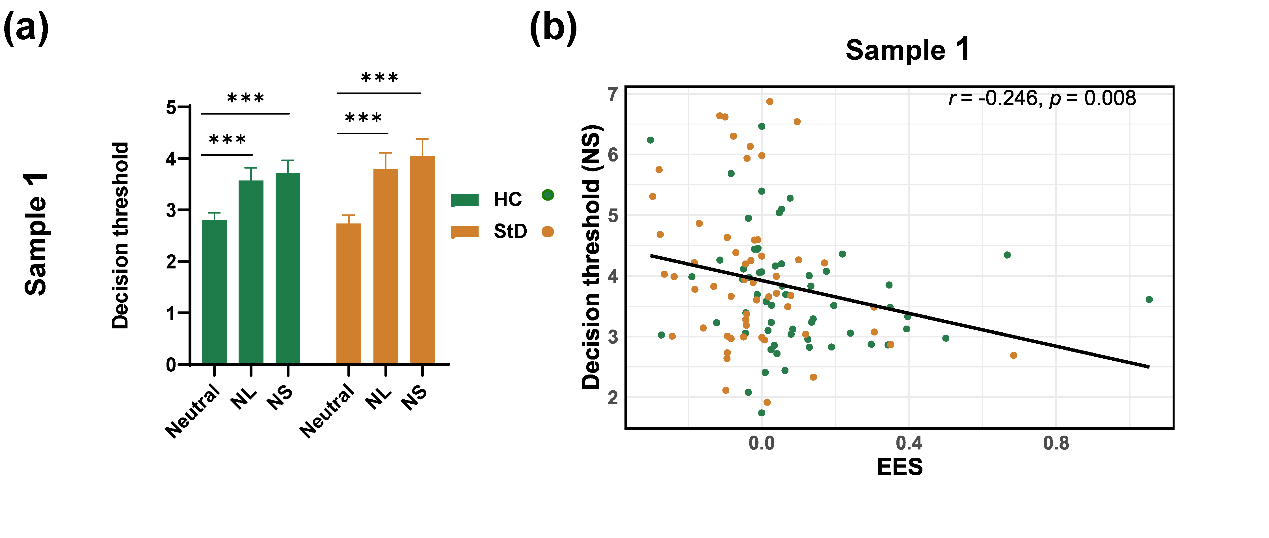


**Fig. S1 Decision threshold in Sample 1. (a)** Both groups had the highest decision threshold in the NS condition. **(b)** There was a negative correlation between EES and Decision threshold in the NS condition. Poorer EES was associated with a higher decision threshold after suppression.


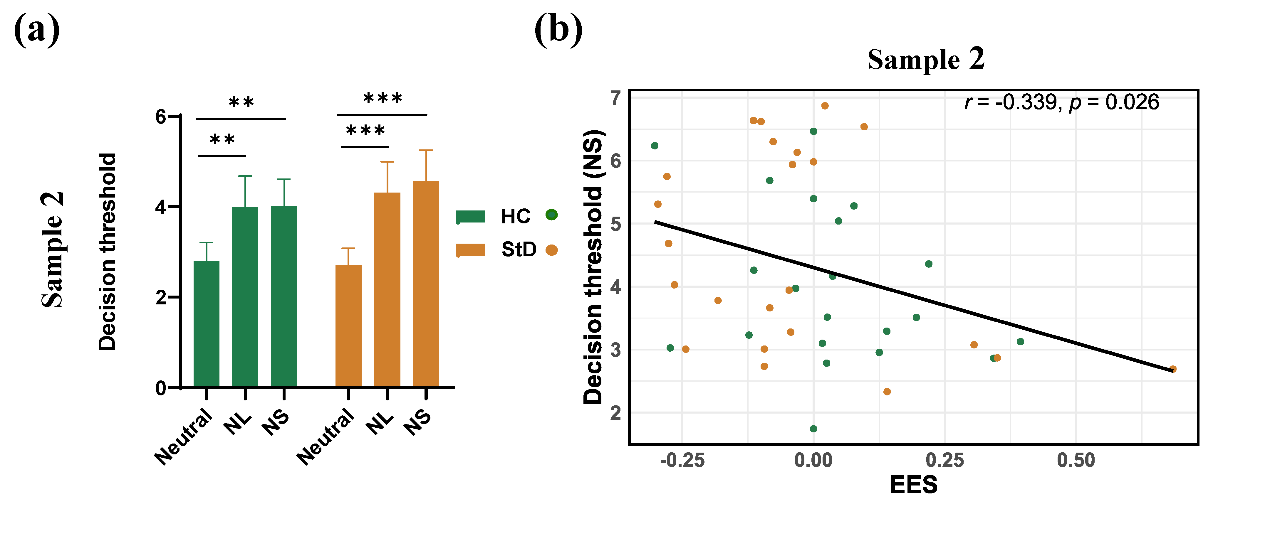


**Fig. S2 Decision threshold in Sample 2.** The results of Sample 2 were consistent with those of Sample 1. **(a)** Both groups had the highest decision threshold in the NS condition. **(b)** There was a negative correlation between EES and Decision threshold in the NS condition. Poorer EES was associated with a higher decision threshold after suppression.


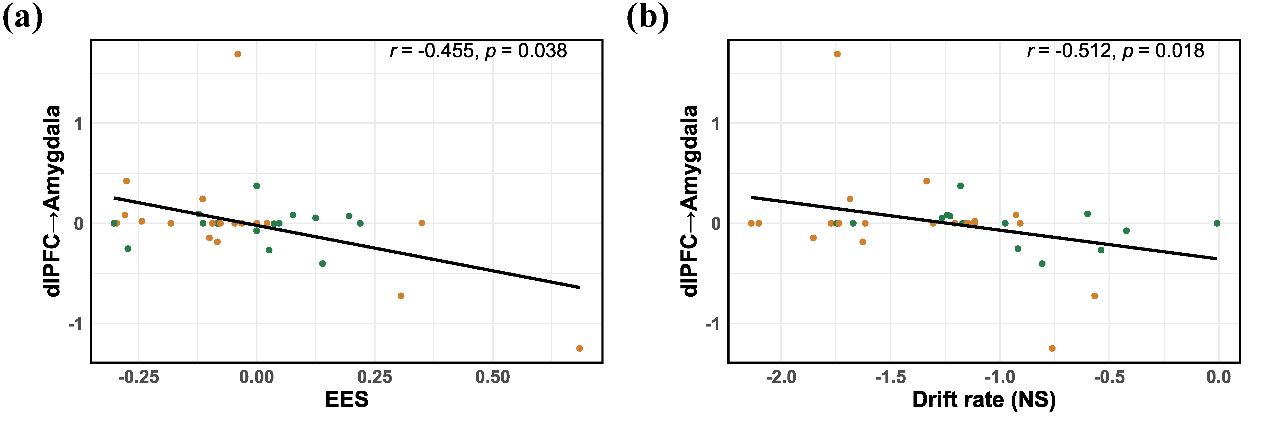


**Fig. S3 The connection strength of the right dlPFC to the right amygdala modulated by NS was extracted for each subject and correlated with EES and drift rate (NS). (a)** The modulated by NS connectivity in right dlPFC to right amygdala connectivity and EES were negatively correlated; **(b)** The modulated by NS connectivity in right dlPFC to right amygdala connectivity and drift rate (NS) were negatively correlated.
